# Preliminary validity testing of the eHealth Literacy Questionnaire (eHLQ): a Confirmatory Factor Analysis (CFA) in Norwegian hospitalized patients

**DOI:** 10.1186/s40359-023-01449-z

**Published:** 2023-11-23

**Authors:** Å. Hermansen, M. H. Andersen, C. R. Borge, K. G. Dahl, M. H. Larsen, K. Lønning, K. B. Meyer, T. K. Vidnes, A. K. Wahl

**Affiliations:** 1 Department of Social Work, Child Welfare and Social Policy, Faculty of Social Sciences, Oslo Metropolitan University, Oslo, Norway; 2 Department of Transplantation Medicine, Oslo University Hospital, Oslo, Norway; 3 Department of Interdisciplinary Health. Institute of Health and Society, Sciences, Faculty of Medicine, University of Oslo, Oslo, Norway; 4 Lovisenberg Diaconale Hospital, Oslo, Norway; 5 Lovisenberg Diaconale University College, Oslo, Norway

**Keywords:** Ehealth literacy, eHealth Literacy Questionnaire (eHLQ), Confirmatory Factor Analysis, Hospitalization

## Abstract

**Aims:**

To perform the first psychometric analysis of the Norwegian version of the eHLQ using confirmative factor analysis (CFA) procedures in a population of patients admitted to hospital using a cross-sectional design. The eHLQ consists of 35 items capturing the 7-dimensional eHealth Literacy Framework (eHLF) which describes users' attributes, user's interaction with technologies and user's experience with digital health systems.

**Methods:**

The 7 independent scales of the eHLQ was translated from Danish and culturally adapted into the Norwegian language following a standardised protocol. Assessment of construct validity of the eHLQ was undertaken using data from a cross-sectional survey of 260 patients hospitalized at a Norwegian University Hospital in the Oslo area during a two-week period in June 2021. The analysis included using correlation analysis (Pearsons R), internal consistency (Cronbach’s alpha) and confirmatory factor analysis (CFA).

**Results:**

All factor loadings were high to acceptable (i.e. > 0.6), except for five items which had somewhat lower loadings. Regarding internal consistency, alpha ranged from 0.73 to 0.90. For optimal CFA fit for the different scale models, correlated residuals were required for five of the seven scales. Overall our analysis shows an intermediate fit of the orginal construct. Scale intercorrelations were all below 0.8, indicating an overall acceptable discriminant validity between the 7 dimensions.

**Conclusions:**

The results from the CFA analysis indicate that for almost all 7 eHLQ scales, an acceptable model fit was achieved. The 260 hospitalized patients included in this study represented a variety of diagnoses, recruited from a geographically limited area. Further studies on psychometric properties of the Norwegian version of eHLQ in larger samples, diverse settings and by using more comprehensive approaches are warranted.

## Background and aim

Electronic health literacy (eHL), is increasingly important as digital services and systems are becoming the norm, and frequently becoming the primary way for people to engage with health services to communicate with healthcare personnel and receive treatments [1, 2]. The importance of evaluating people’s eHL has accelerated as health professionals seek to adapt digital health services to patient care, including forms of virtual care, remote monitoring, artificial intelligence, smart wearables across a range of digital platforms. The modern health care system brings increased complexity for patients and people in the community. For health care users to be able to effectively and equitably access health services, it is critical that eHL becomes a focus for health care services development and research [1]. This is aligned with WHO`s global strategy on digital health 2020–25 [3] which aims to “Strengthen health systems through the application of digital health technologies for consumers, health professionals, health care providers and industry towards empowering patients and achieving the vision of health for all” [3].

Although there are several definitions of eHL [4], the concept may be defined as “a dynamic and context-specific set of individual and social factors, as well as consideration of technological constraints in the use of digital technologies to search, acquire, comprehend, appraise, communicate, apply and create health information in all contexts of healthcare with the goal of maintaining or improving the quality of life throughout the lifespan” [4]. There also exist several theoretical frameworks to explain eHL and eHL measures [4–6]. One of the most recognized frameworks is the eHealth Literacy Framework (eHLF) [6]. eHLF describes the attributes of the users (information and knowledge about their health); the intersection between users and the technologies (their feeling of being safe and in control and their motivation); and users’ experience of systems (how they work and are accessible, and suits users’ needs). The eHLF was specifically designed to inform the development of a conceptually and psychometrically sound questionnaire, that is, the eHealth Literacy Questionnaire (eHLQ) [1].

## Previous research

Six previous studies have investigated the validity of the eHealth Literacy Questionnaire using both confirmative factor analysis [1, 7, 8] and Bayesian structural equation modelling [9, 10]. The rationale for focusing on the eHLQ was that this instrument is multi-dimensional, based on a well-defined theoretical framework, and has showed to be psychometrically robust when used in conditions comparable to Norwegian conditions.

A recent review of ehealth literacy instruments and their measurement properties identified the seven instruments used to measure ehealth literacy: eHealth literacy scale (eHEALS), eHealth literacy scale–extended (eHEALS-E), electronic health literacy scale (e-HLS), digital health literacy instrument (DHLI), eHealth literacy assessment toolkit (eHLA), eHealth literacy questionnaire (eHLQ), and transactional eHealth literacy instrument (TeHLI). The review concluded that further psychometric studies are warranted [5].

Development of the eHLQ followed the validity-driven approach which has been used to develop several widely used and highly robust questionnaires [6, 11, 12]. Kayser et. al. tested the items based on a sample of 475 people recruited in community and health care settings and including people with a range of chronic conditions [1]. Chen et. al. included a random sample of 442 Chinese adults attending outpatient departments at several hospitals in Taiwan [9]. Whereas Cheng et. al. recruited 525 participates from three health sites in Victoria, Australia, in 2018 [10, 12].

Along with the evolution of interactive communication technologies of the internet, conceptual extensions have been demanded for eHL. This has resulted in the development of second-generation instruments, among those the eHLQ, to measure a wider range of eHealth literacy concepts to make them more suitable for people living in the social-media era of eHealth. However, those instruments have up to now been assessed in a limited number of studies and synthesized evidence for the measurement properties are lacking. Therefore, further studies are strongly recommended [5]. The present paper reports the first psychometric analysis of the Norwegian version of the eHLQ using confirmative factor analysis (CFA) procedures in a population of patients admitted to hospital.

### Design

Using a cross-sectional design, questionnaires were administered to 260 adults admitted across four clinical departments at a Norwegian University Hospital in the Oslo area during a two-week period in June 2021. The clinical areas are grouped into four divisions: Department of Gastrointestional and Children Surgery, Department of Urology, Department of Transplantation, and Department of Rheumatology, Inflammatory Disorders and Skin diseases.

We translated the Danish version of the questionnaire into Norwegian through a translation company. Since the Norwegian and Danish languages are similar understood by the research team, we did not back translate to Danish. The research group included four researchers from Norway and one that spoke and wrote Danish. We also examined the English version when generating the draft forward translation and this version was applied in 5 cognitive interviews.

### Setting

This study was performed in Norway where there exists no compulsory private insurance, so everyone is entitled to free healthcare under the public system. Healthcare in Norway is mostly provided by the government through municipal health services. Every citizen and resident of Norway is entitled to healthcare and the government ensures that citizens receive an equal standard of care at all hospitals.

### eHealth Literacy Questionnaire (eHLQ)

The objective of the eHLQ development was to create an instrument that captures the 7 hypothesized dimensions of eHLF. The eHLQ was developed in Danish and English concomitantly to assist with identifying and removing idiomatic expressions and to support accurate adaptation to other languages. The eHLQ has been shown to be a psychometrically robust multidimensional instrument with 35 items and 7 scales that comprehensively cover all 7 dimensions of the eHLF [6, 11, 12]. Hence, the eHLQ and the eHLF’s conceptual underpinnings are likely to be a useful set of tools to support researchers, developers, designers, and governments to develop, implement, and evaluate effective digital health interventions and to identify vulnerable groups in the health care setting.

The eHLQ seeks to support researchers, developers, designers, and governments to develop, implement, and evaluate effective digital health interventions [1]. The eHLQ consists of seven scales: 1: Using technology to process health information (5 items), 2: Understanding of health concepts and language (5 items), 3: Ability to actively engage with digital services (5 items), 4: Feel safe and in control (5 items), 5: Motivated to engage with digital services (5 items), 6: Access to digital services that work (6 items) and 7: Digital services that suit individual needs (4 items). Each item is scored using 4-point ordinal scale response options from strongly disagree (1) to strongly agree (4). The presence of the 7- factor structure and supportive validity evidence has been confirmed in previous studies [1, 8–10].

### Sample

The majority of patients had chronic conditions and were dependent of long-term interactions with multidisciplinary health care providers. The following inclusion criteria were: Being 18 years or older, hospitalized during a two-week period, and speaking and understanding Norwegian language. Patients fulfilling the inclusion criteria were consecutively contacted by a study nurse, provided with information about the study, and if they were interested in taking part, provided a written informed consent. All new patients that were hospitalized at one of the four unites during the two week period were invited to participate. All patients answered on pen -and-paper questionnaires while at the hospital without assistance from the health care personal.

In addition to eHLQ data, the present paper draws on self-reported data on sociodemographic information (age, sex, marital status, education, and work status), diagnoses and self-reported health using EQ-5D. The EQ-5D is a generic questionnaire measuring five different dimensions of health (mobility, self-care, usual activities, pain/discomfort and anxiety/depression). It contains five simple questions, and a total score of one´s own health in the end, a VAS score of 1–100 where 1 represents the worst possible measure of health and 100 the best possible health measure [13]. We used the VAS part from the EQ-5D as this part captures the respondent's overall rating of their health.

### Data analysis

We use confirmatory factor analysis (CFA). CFA is a type of structural equation modelling (SEM) employed to validate the factor arrangement within a given set of observed variables. CFA enables us to assess the hypothesis that there is an established connection between the observed items and the concealed latent constructs of the 7 hypothesized dimensions of eHLF [14].

Analyses were carried out using Stata v17 (StataCorp, College Station, TX). Descriptive analyses were carried out to characterise the sample. Further, to investigate each scale’s internal consistency, Cronbach’s alpha was calculated. A Cronbach’s alpha of 0.70 and above is generally considered as indicating an acceptable internal consistency in the responses for the items included in a scale, thus indicating that the included items measure the same underlying dimension. Alpha assesses the extent to which items produce consistent scores and should be understood as the fraction of measurement variability associated with variations in an individual's actual score range [15].

CFAs were performed in order to confirm the factorial structure of the questionnaire. The factor structure was specified a priori, given that the structure of the original eHLQ was described previously [1]. Consequently, confirmatory analyses were done exclusively. For model estimation, maximum likelihood was applied as estimator.

First, seven one factor models were fitted to the data to investigate the factor loadings of each item. Correlated residuals were sequentially added to respective models when fitting the one-factor models for each of the seven scales. To test whether these modifications, in terms of correlated within-factor residuals, led to significant model improvement, modification indices were obtained using the ‘estat mindices’ command in Stata. To investigate discriminant validity intercorrelations between scales were investigated by using Pearson r.

Model evaluation was based on chi-square tests for model fit and further model fit indices, including the root mean square error of approximation (RMSEA), the comparative fit index (CFI), the Tucker–Lewis index (TLI) and the standardised root mean square residual (SRMR). For model fit to be interpreted as ‘acceptable’, an RMSEA of < 0.05 was considered a close fit, while an RMSEA and an SRMR of up to 0.08 were considered acceptable. Comparing the fit of a target model to the fit of an independent or null model, the CFI has a cut-off for good fit CFI of ⩾0.90. A TLI of 0.95 indicates the model of interest improves the fit by 95% relative to the null model, and the cut-off for good fit was set at TLI ⩾0.95. Furthermore, the correlations of residuals to improve model fit when fitting the seven one-factor models were considered. Correlated residuals, a partial correlations between the unexplained variance from two items, of < 0.2 were considered acceptable when fitting the models [16, 17]. Potential model adjustments for improved fit were based on modification indices as provided in the Stata output using the ‘estat gof, stats (all)’ command.

## Results

Sample characteristics of the 260 respondents are shown in Table 1. The sample consisted of 41% women. Age ranged from 19 to 89 years, with a mean age of 55 years (*SD* = 16.3 years), 40% had Secondary/High school as their highest level of education, wereas 24% reported to have finished a higher education of 4 years or less and 28 percent reported to have a higher education of 4 yers or more. Almost half, 48%, reported to be employed and 40% were on disability benefits or retired. The great majority, 70%, reported to be married or cohabitant. Almost three in ten, 29%, were hospitalized due to cancer, 12% were kidney related and 8% did not disclose wich diagnosis they had. Mean self-assessed health was 60 (range 0 to 100, with a higher number indicating better health).

**Table 1:** Characteristics of the study-population (*n* = 260)

	N/%
Sex (biological)	
Women	106/41%
Men	154/59%
Educational level	
Primary school	14/5%
Secondary/High school	103/40%
College/university < 4 years	62/24%
College/university ≥ 4 years	72/28%
Missing	9/3%
Work status	
Employed	125/48%
Disability benefits or retired	104/40%
Student	8/3%
Unemployed	6/2%
Other	12/5%
Missing	5/2%
Civil status	
Married or co-habitant	181/70%
Single/divorced/separted	53/20%
Widow/widower	12/4%
Missing	14/5%
Self-reported diagnose	
Cancer	76/29%
Kidney	31/12%
Bile	11/4%
Liver	10/4%
Prostate	8/3%
Systemic sclerosis	6/2%
Other	98/38%
Missing	20/8%
	Mean (min–max) SD^a^
Age	55 (19–89) 16.300
Self-reported health (VAS)^b^ (range 0–100)	60.1 (0–95) 20.700

^a^*SD* Standard deviation, ^b^*VAS* Visual analog scale

Seven one factor models were fitted to the data (see Table 2). For each scale, there were high to acceptable loadings on all items (i.e. > 0.6; see column ‘Standardised factor loading’ in Table 2), except for two items in scale 2 (Understanding of health concepts and language) eHLQ15 (0.56) and eHLQ26 (0.34), one item in scale 4 (Feel safe and in control), eHLQ14 (0.51), and two items in scale 6 (Access to digital services that work); eHLQ3 (0.58) and eHLQ23 (0.51). In total five items out of 35 (14.2%), representing three scales, were identified and had factor loadings below 0.6.

**Table 2:** Confirmatory factor analysis of the eHLQ – seven one factor models

Scale/item number	Standarised factor loading	Standard error
1. Using technology to process health information		
EHLQ7	0.824	0.028
EHLQ11	0.842	0.027
EHLQ13	0.660	0.041
EHLQ20	0.657	0.042
EHLQ25	0.708	0.038
2. Understanding of health concepts and language		
EHLQ5	0.734	0.042
EHLQ12	0.765	0.040
EHLQ15	0.564	0.053
EHLQ21	0.664	0.046
EHLQ26	0.347	0.065
3. Ability to actively engage with digital services		
EHLQ4	0.763	0.031
EHLQ6	0.874	0.021
EHLQ8	0.749	0.032
EHLQ17	0.823	0.026
EHLQ32	0.810	0.027
4. Feel safe and in control		
EHLQ1	0.768	0.034
EHLQ10	0.697	0.039
EHLQ14	0.512	0.053
EHLQ22	0.823	0.030
EHLQ30	0.721	0.038
5. Motivated to engage with digital services		
EHLQ2	0.660	0.043
EHLQ19	0.801	0.032
EHLQ24	0.645	0.045
EHLQ27	0.776	0.034
EHLQ35	0.742	0.036
6. Access to digital services that work		
EHLQ3	0.581	0.056
EHLQ9	0.600	0.053
EHLQ16	0.607	0.053
EHLQ23	0.519	0.059
EHLQ29	0.666	0.049
EHLQ34	0.622	0.053
7. Digital services that suit individual needs		
EHLQ18	0.717	0.039
EHLQ28	0.800	0.033
EHLQ31	0.765	0.035
EHLQ33	0.754	0.036

When fitting the one-factor models for each of the seven scales, correlated residuals were sequentially added to respective models, which improved each model fit significantly. Table 3 shows the results of the CFA separately for the seven individual eHLQ scales. For optimal CFA fit for the different scale models, correlated residuals were required for five of the seven scales. For one of the scales (scale 1: Using technology to process health information), three correlated residuals were required to acquire an acceptable CFA model fit. For two of the scales (scale 2: Understanding of health concepts and language and scale 6: Access to digital services that work), an acceptable CFA model fit was obtained after two correlated residuals. Two of the scales (scale 3: Ability to actively engage with digital services and scale 5: Motivated to engage with digital services) had an acceptable CFA model fit after one correlated residual. Lastley, two of the scales (scale 4: Feel safe and in control and scale 7: Digital services that suit individual needs) had an acceptable model fit without adjustments. Internal consistency (Cronbach’s alpha) ranged from 0.73 (scale 2: Understanding of health concepts and language) to 0.90 (scale 3: Ability to actively engage with digital services).

**Table 3:** Confirmatory factor analysis and internal consistency (Cronbach’s alpha) of individual scales of the eHLQ

Model	X^2^	p	RMSEA	CFI	TLI	SRMR	Correlated error
**Scale 1: Using technology to process health information**							
Original	25.650	0.000	0.130	0.962	0.923	0.038	
EHLQ7 with EHLQ13	3.470	0.176	0.055	0.997	0.986	0.015	-.085
EHLQ20 with EHLQ25							-.051
EHLQ13 with EHLQ25							.059
Cronbach`s alpha: 0.85							
**Scale 2: Understanding of health concepts and language**							
Original	21.280	0.001	0.115	0.945	0.889	0.051	
EHLQ5 with EHLQ26	1.580	0.664	0.000	1.000	1.016	0.012	.065
EHLQ15 with EHLQ26							.092
Cronbach`s alpha: 0.73							
**Scale 3: Ability to actively engage with digital services**							
Original	35.600	0.000	0.159	0.959	0.918	0.034	
EHLQ17 with EHLQ32	6.080	0.193	0.046	0.997	0.993	0.015	.101
Cronbach`s alpha: 0.90							
**Scale 4: Feel safe and in control**							
Original	9.180	0.102	0.059	0.990	0.981	0.026	
Cronbach`s alpha: 0.83							
**Scale 5: Motivated to engage with digital services**							
Original	31.690	0.000	0.149	0.945	0.890	0.045	
EHLQ24 with EHLQ27	7.430	0.115	0.060	0.993	0.982	0.019	.091
Cronbach`s alpha: 0.84							
**Scale 6: Access to digital services that work**							
Original	50.160	0.000	0.139	0.872	0.787	0.062	
EHLQ3 with EHLQ23	12.470	0.086	0.058	0.983	0.964	0.032	.092
EHLQ3 with EHLQ23							-.134
Cronbach`s alpha: 0.76							
**Scale 7: Digital services that suit individual needs**							
Original	1.350	0.508	0.000	1.000	1.005	0.010	
Cronbach`s alpha: 0.84							

The correlations between scales are shown in Table 4. The highest correlations were between scale 1 (using technology to process health information) and scale 3 (ability to actively engage with digital services); 0.797 and scale 5 (motivated to engage with digital services) and scale 7 (Digital services that suit individual needs); 0.792. The lowest correlation was between scale 3 (ability to actively engage with digital services) and scale 4 (feel safe and in control); 0.296. These results showed an overall acceptable discriminant validity between the 7 dimensions. As shown in Table 4, age was negatively correlated with all scales, except for scale 4 (feel safe and in control) and scale 6 (access to digital services that work). There were no differences according to sex. Those who are employed generally scored higher on all scales, except for scale 5 (motivated to engage with digital services), 6 (access to digital services that work) and scale 7 (Digital services that suit individual needs).

**Table 4:** Correlation between the seven domains included in the eHLQ and selected background characteristics

	1. Using technology to process health infor- mation	2. Under- standing of health concepts and language	3. Ability to actively engage with digital services	4. Feel safe and in control	5. Motivated to engage with digital services	6. Access to digital services that work	7. Digital services that suit individual needs	Age	Woman	Higher education	Employed	Disability benefits or retired	Married or co-habitant	Cancer	Self-reported health (VAS)
1. Using technology to process health information	1														
2. Understanding of health concepts and language	0.596***	1													
3. Ability to actively engage with digital services	0.797***	0.582***	1												
4. Feel safe and in control	0.318***	0.426***	0.296***	1											
5. Motivated to engage with digital services	0.792***	0.541***	0.622***	0.352***	1										
6. Access to digital services that work	0.545***	0.415***	0.458***	0.497***	0.548***	1									
7. Digital services that suit individual needs	0.690***	0.461***	0.627***	0.367***	0.729***	0.702***	1								
Age	-0.285***	-0.156**	-0.382***	-0.051	-0.234***	-0.096	-0.262***	1							
Woman	-0.017	-0.016	-0.077	-0.004	-0.062	-0.003	-0.039	-0.118	1						
Higher education	0.249***	0.289***	0.287***	0.108	0.196**	0.071	0.119	-0.065	-0.025	1					
Employed	0.323***	0.205***	0.337***	0.133**	0.249***	0.103	0.214***	-0.369***	-0.140**	0.301***	1				
Disability benefits or retired	-0.309***	-0.189***	-0.317***	-0.115	-0.227***	-0.095	-0.193***	0.533***	0.057	-0.245***	-0.786***	1			
Married or co-habitant	0.098	0.055	0.062	0.048	0.067	0.070	-0.008	0.089	0.003	0.112	0.134**	0.012	1		
Cancer	-0.014	0.004	-0.108	-0.038	-0.050	-0.050	-0.015	0.326***	-0.137	0.065	-0.043	0.131	0.112	1	
Self-reported health (VAS)	0.074	0.098	0.143**	0.161**	0.086	0.172***	0.109	0.067	-0.149**	0.083	0.118	-0.044	0.069	-0.031	1
**: *p* ≤ 0.05, ***: *p* ≤ 0.01															

## Strengths and limitations

The present paper reports the first psychometric analysis of the Norwegian version of the eHLQ using confirmative factor analysis (CFA) procedures in a population of patients admitted to hospital. The sample size is rather small and the 260 hospitalized patients included in this study is recruited from a geographically limited area.

## Discussion

We found the eHLQ to have a moderate fit when interpreting CFA results, internal consistency of each of the seven domains and intercorrelations between scales. Our study has provided further evidence to support the internal consistency of the eHLQ and also moderate support regarding structural validity.All scales in our study had a α > 0.80, except scale 2 with a α = 0.73 and scale 6 with a α = 0.76.. In a study by Chen et. al., seven one-factor models for each of the seven scales were fitted and the results showed that the models generally fitted the data well for all scales, except for scale 2 (Understanding of health concepts and language) for which correlated residuals were needed to get a good fit [1]. Compared with our results, Chen et. al. obtained models that generally fitted the data better, requiring fewer modifications using correlated residuals and higher factor loadings (all loadings were > 0.50).

In the last couple of years, several papers have been published further informing the evidence of the validity and reliability of the eHLQ [8–10]. In their review study, Cheng et. al. found found evidence of satisfactory – to – strong psychometric properties of the eHLQ [10]. They concluded that the seven eHLQ scales are likely to be useful research tools for evaluating digital health interventions and for informing the development of health technology products and interventions that equitable suit diverse users’ needs in the Mandarin language. Furthermore, Cheng et. al. [10] found, by using a Bayesian mediated multiple indicators multiple causes model, supportive validity evidence for the eHLQ based on relations to other variables. In the Australian community health context, Cheng et. al. established evidence regarding the internal structure related to measurement invariance across the groups for the 7 scales. In another study by Cheng et. al. [12], using a mixed methods approach for validity testing, the results suggest that the eHLQ is a tool with robust psychometric properties regarding internal structure, but further investigation of discriminant validity is recommended. They claim that the eHLQ is ready to be used to identify e-health literacy strengths and challenges and assist the development of digital health interventions to ensure that people with limited digital access and skills are not left behind. With significant lower scores on several scales, our results show that those with lower education and also those of higher age may need special attention regarding skills and motivation.

In a recent study reporting on the translation and validation of the Swedish version of the eHLQ in 236 primary health care patients and parents of hospitalzed children, all the seven eHLQ scales showed good internal consistency and satisfactory model-fit values [18]. With one exception, all items demonstrated satisfactory loadings on their respective factors. In our study five items had low factor loadings (i.e. < 0.6), however internal consistency estimates were satisfactory.

In our study, five of the scales needed correlated residual adjustments to acquire optimal model fit. Especially scale 1 – using technology to process health information (three corrections), scale 2 – understanding of health concepts and language (two corrections), and scale 6 – access to digital services that work (two corrections) needed more than one correction. Item factor loadings were high to acceptable (above 0.6) for almost all items, except for four of the items with loadings at a 0.5 level which could be seen as acceptable and one item with a loading of 0.347. These findings are somewhat comparable to the findings in the studies by Cheng et al. [10]. In the study by Cheng et al. [10] all scales had a composite scale reliability ranging from 0.73 to 0.90, which was the same as in our study. Furthermore, in the study by Cheng et al. [10] a Bayesian structural equation modelling was used, and the model of interest produced a satisfactory fit. All items loaded on the relevant factors, with loadings from 0.36 to 0.94 [10].

The eHLQ was developed using both classical and modern psychometric approaches to questionnaire development. The eHLQ is also based on a well-defined a priori eHLF framework which strengthens the instruments’ construct and content validity. In our study, we used a classical approach (CFA) to gain evidence for the eHLQ psychometric robustness. However, by taking modern psychometric approaches into use in the validation process of the Norwegian version of the eHLQ, further evidence could be established regarding construct and other types of validity. In addition, using more comprehensive methods in psychometric evaluation, will add important information about the eHLQ with regard to meaningfulness and relevance [19]. The setting for our study was hospitalized patients, although with a variety of diagnosis and ages, the sample size was modest, including 260 patients. Although the eHLQ (English, Danish and Chinese version) shows robust results internationally [1, 8–10], further testing of the Norwegian version of the eHLQ in warranted, such as testing the instrument based on larger samples, but also testing it at local hospitals in Norway and in community based settings.

## Conclusions

A classical psychometric approach, applied to the Norwegian version of the eHLQ, have provided preliminary evidence that the questionnaire is likely to be a useful eHL tool in a hospital setting in Norway. Additional psychometric investigations, in other healtcare settings and including other populations with a greater geographical coverage, are needed to strengthen the evidence for validity and reliability of data collected using the eHLQ in a Norwegian context.

## Data Availability

The data that support the findings of this study were used under license for the current study, and so are not publicly available.

## Abbreviations

eHLQ: EHealth Literacy Questionnaire
CFA: Confirmative factor analysis
RMSEA: Root mean square error of approximation
CFI: Comparative fit index
TLI: Tucker-Lewis index
SRMR: Standardized root mean square residual
P: Probability
